# Acute onset polyarthritis in older people: Is it RS3PE syndrome?

**DOI:** 10.1186/1757-1626-1-132

**Published:** 2008-08-29

**Authors:** Abdul Salam, Rafik Henry, Tom Sheeran

**Affiliations:** 1 Department of Geriatric Medicine, Good Hope Hospital, Rectory Road, Sutton Coldfield, UK; 2 Department of Rheumatology, Mid Staffordshire General Hospital, Cannock Chase Hospital, Brunswick Road, Cannock, UK

## Abstract

Remitting Seronegative Symmetrical Synovitis with Pitting oedema syndrome, a rare inflammatory arthritis, commonly affects people in the older age group. It can present as an acute onset polyarthritis with associated pitting oedema of the extremities. Patients show excellent response to low dose steroids with complete and sustained remissions. It can also be a paraneoplastic manifestation of an underlying occult malignancy, hence thorough clinical evaluation is warranted.

We discuss a case of Remitting Seronegative Symmetrical Synovitis with pitting oedema syndrome where the patient presented with acute onset polyarthritis and pitting oedema of the extremities without an underlying systemic cause. Patient showed dramatic response to low dose steroids.

## Introduction

Remitting Seronegative Symmetrical Synovitis with Pitting oedema (RS3PE) syndrome, a subset of acute onset polyarthritis mainly affects the older people and predominantly males with clinical manifestations of acute onset pitting oedema of the hands. Other notable features include seronegativity for Rheumatoid factor and an excellent response to low dose steroids with long-term remission.

In 1985, McCarty et al. [1] described the first case of Remitting Seronegative Symmetrical Synovitis with Pitting Oedema (RS3PE) syndrome characterized by symmetrical distal synovitis and tenosynovitis of the mucous sheaths of the flexor and extensor tendons of the hands associated with pitting oedema of the hands and/or feet.

In this case report we discuss a case of RS3PE syndrome where the initial presentation was bilateral pitting oedema of the extremities without any other systemic cause. Patient showed dramatic response to low dose steroids.

## Case presentation

A 67-year-old male Caucasian patient was admitted with a 2-week history of painful swollen hands and painful knees associated with worsening mobility. This was preceded by a history of swinging low-grade pyrexia for two months and a history of progressive pedal oedema for 4 months. The symptoms were atraumatic in onset and lacked any associated features of connective tissue disease. There was a positive past medical history of systemic hypertension, hypothyroidism, diabetes mellitus, chronic renal failure and Parkinsons disease. The patient had been treated for carcinoma of the prostate gland in the past.

Examination revealed bilateral pitting oedema of dorsum of hands and legs upto the calves. He also had synovitis at proximal interphalangeal joints, wrists and effusion of both knees and ankles. Initial blood test showed haemoglobin of 9.5 g/l with normochromic and nomocytic anaemia, raised inflammatory markers (ESR 70, CRP 100) and normal WBC. Autoantibody screen and rheumatoid factor were negative. Radiological findings of hands, feet and knees did not show any erosions. The patient was also screened and investigated for associated malignancies. He had normal tumour markers including CEA, AFP, CA19-9 and PSA (Prostrate specific antigen). CT thorax/abdomen and OGD were also reported as normal. In view of low-grade pyrexia, possibility of infective focus was ruled out by repeated blood and urine cultures. A diagnosis of remitting symmetrical seronegative synovitis with pedal oedema was suggested and patient responded extremely well to low dose prednisolone at 7.5 mg daily dosage. Further follow up 8 weeks later on tapering dose of prednisolone showed complete resolution of signs and symptoms without any further flare-ups.

## Discussion

In 1985, Mc Carty [1] described first RS3PE syndrome in a series of 10 patients. Following a retrospective multicenter study of patients Olive *et al*. [2] proposed the following diagnostic criteria for this syndrome:

1. Bilateral pitting oedema of both hands

2. Sudden onset of polyarthritis

3. Age more than 50 years

4. Seronegative rheumatoid factor.

The exact incidence and prevalence are not known. RS3PE affects more men than women with ratio of 2:1 and more frequently the older people.

The aetiology and the pathogenic mechanisms are not clear. The syndrome was reported to be associated with HLA B7 [1] and HLA A2 haplotype [3].

Recently, vascular endothelial growth factor (VEGF) has been implicated as a contributing factor for pathological changes responsible for both hypervascularity (synovitis) and vascular permeability (subcutaneous oedema) [4].

Aetiology of pitting oedema is not known, but recent MRI studies suggest that marked extensor tenosynovitis is the principle lesion responsible for oedema of subcutaneous and peritendinous soft tissue [5].

Fever and asthenia could be non-specific manifestations of inflammation, but presence of other systemic signs like weight loss, anorexia and poor response to steroids could indicate a paraneoplastic manifestation.

Evaluation for pedal oedema should aim at ruling out the other possibilities like congestive cardiac failure, nephritic syndrome and hypothyroidism.

In older people it is important to distinguish this syndrome from PMR (Polymyalgia Rheumatica) in view of the duration of treatment with steroids that is needed. This is even more pertinent in view of the long-term consequences of the use of steroids in older people, as these patients are more likely to be already having multiple co morbid factors like osteoporosis, hypertension, diabetes and heart failure. RS3PE syndrome can be associated with both solid tumours like gastric [6], pancreatic [7] and haematological malignancies like non-hodgkin's lymphoma [8].

Patients with idiopathic RS3PE showed an excellent response to low doses of corticosteroids compared to the poor response to RS3PE in association with neoplasia [9].

Clinicians need to be aware of the RS3PE in ageing population and initiate appropriate investigations to exclude any occult malignancy. The search for occult malignancy is particularly crucial in patients whose systemic symptoms are prominent and who are still not responsive to steroids.

The main differential diagnosis of RS3PE is polymyalgia rheumatica, which can be very difficult in older people. The features helpful in differentiating the two can be seen in Table 1.

**Table 1:** Differentiating features of RS3PE Syndrome and PMR

**RS3PE Syndrome**	**PMR**
Dramatic response to low dose steroids or NSAIDS and sometimes to hydroxychloroquine.	Responds only to steroids

Common in males	More frequent in females

Mainly involves wrist with pitting oedema.	Involves shoulder and pelvic girdle with associated systemic symptoms. Pitting oedema present rarely.

Association with HLA-B7, B27, A2	HLA association with HLA-DR4

Excellent long-term prognosis	Frequent relapses and recurrences.

Other differential diagnosis with RS3PE in older people includes Rheumatoid arthritis, late onset spondyloarthropathy, mixed connective tissue disease, chondrocalcinosis and amyloid arthropathy.

Blood tests may typically demonstrate raised inflammatory markers, discrete inflammatory anaemia, and a negative rheumatoid factor. X-rays of the hands and wrists may show soft tissue swelling but absence of erosions is classic. Tenosynovitis of both flexor and extensor tendons at the wrist and the extensor tendons of the feet is the hallmark of RS3PE. Ultrasonograph a reliable and cost effective modality for evaluation of patients with suspected RS3PE this characteristically shows tenosynovitis of flexor and extensor tendons [10].

MRI is useful for monitoring the disease activity in RS3PE syndrome. It can also provide information about soft tissue, cartilage and bony erosions [11].

Whole body Ga-67 scan can show increased uptake in hands and feet and this could be useful in assessing lesion activity [12]. Importantly appropriate investigations need to be carried out if there is any suspicion of malignancy.

Nonsteroidal anti-inflammatory drugs or Salicylates for pain relief. Most of the patients respond very well to low dose of steroids (10–15 mg prednisolone) and these patients show sustained and complete remission even after withdrawal of steroids. Some patients respond to hydroxyl chloroquine or gold salts.

## Conclusion

RS3PE is a definite syndrome, subset of polyarthritis with favourable outcome and has a good prognosis in the older patients. It may present, as a paraneoplastic manifestation especially in older people who show a poor response to steroids. If suspected, looking for underlying malignancy is recommended.

## Abbreviations

RS3PE: Remitting seronegative symmetrical synovitis with pitting oedema, NSAID: Nonsteroidal anti-inflammatory drug, MRI: Magnetic resonance imaging, HLA: Human leukocyte antigen, PMR: Polymyalgia Rheumatica, WBC: White blood cell, ESR: Erythrocyte sedimentation rate, CRP: C-reactive protein, CEA: Carcinoembryonic antigen, AFP: Alpha-fetoprotein, CA19-9: Carbohydrate antigen 19-9, PSA: Prostate specific antigen, VEGF: Vascular endothelial growth factor.

## Competing interests

The authors declare that they have no competing interests.

## Authors' contributions

AS was involved in the initial drafting and formatting of manuscript. RH and TS revised and corrected the manuscript. All authors have read and approved the final manuscript.

## Consent

Written informed consent was obtained from the patient for publication of this case report and accompanying images. A copy of the written consent is available for review by the Editor-in-Chief of this journal ' [see Additional file 1]'.

## Supplementary Material

Additional File 1 Consent form page 1 Click here for file
