# Supplementary figures and images for: Acute onset polyarthritis in older people: Is it RS3PE syndrome?
Source: Cases J. 2008 Aug 29;1:132. doi: 10.1186/1757-1626-1-132 (PMC2543002; doi:10.1186/1757-1626-1-132)

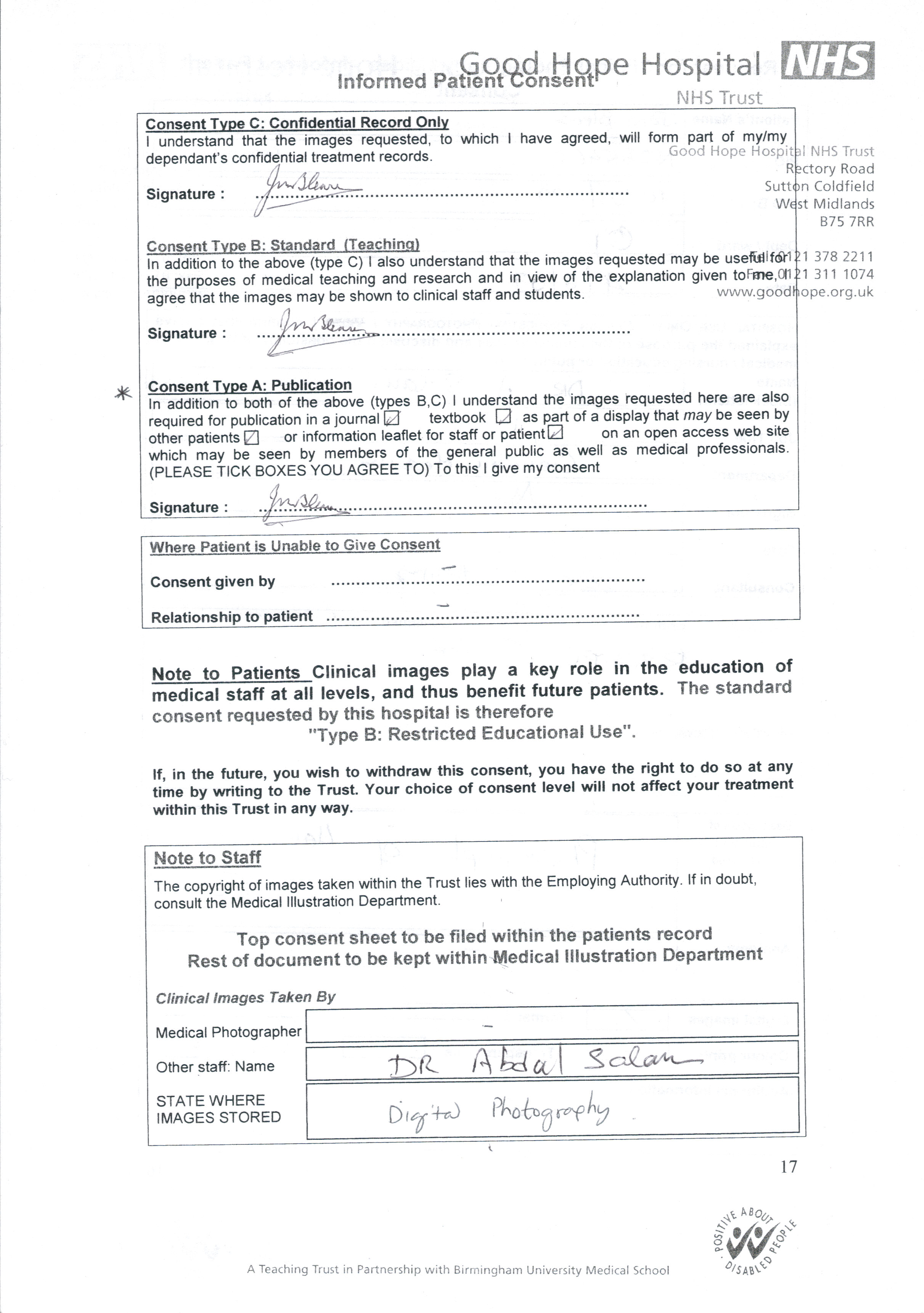

Supplement: Additional File 1 — Consent form page 1 [file 1757-1626-1-132-S1.jpeg]
